# Complete mitochondrial genome sequence of *Glaucosoma buergeri* (Pempheriformes: Glaucosomatidae) with implications based on the phylogenetic position

**DOI:** 10.1080/23802359.2018.1424583

**Published:** 2018-01-10

**Authors:** Takashi P. Satoh

**Affiliations:** Seto Marine Biological Laboratory, Field Science Education and Research Center, Kyoto University, Nishimuro, Wakayama, Japan

**Keywords:** Glaucosomatidae, Pempheriformes, mitogenome, *Glaucosoma buergeri*

## Abstract

The complete mitochondrial genome sequence of pearl perch (*Glaucosoma buergeri*) was determined using a PCR-based method. The genome was 16,529 bp in length and included 37 genes (13 protein-coding genes, 22 transfer RNA genes, and two ribosomal RNA genes) and two non-coding regions (control region and origin of the light strand replication). A maximum likelihood analysis was conducted to confirm the phylogenetic position of this species using almost all the data available on Pempheriformes in the database. The complete mitochondrial genome sequence data obtained from our study would be useful for analyzing the evolutionary relationships of the Pempheriformes and population genetics of the Glaucosomatidae.

The Glaucosomatidae is a small monogeneric family comprising of four species, three of which are endemic to Australia, and one (*Glaucosoma buergeri*) is widely distributed from Western Australia to Southern Japan (Mckay 1997). Glaucosomatids are of commercial and recreational value in these areas. Therefore, molecular data of these species are indispensable for their resource management and conservation. However, the genetic information on glaucosomatids is insufficient as only partial genomic or mitochondrial DNA sequences of approximately 1000 bp have been registered in public databases. In the present study, we determined the complete mitogenome sequence of *G. buergeri* and conducted phylogenetic analysis including Pempheriform fishes that have been shown to be closely related to the glaucosomatids based on previous studies (Mckay 1997; Near et al. 2013).

A specimen of *G. buergeri* was purchased from the Kyoto City Central Wholesale Market in Kyoto Prefecture, Japan, and deposited at the National Museum of Nature and Science, Tokyo (Voucher number: NSMT-P 101224). The complete mitogenome of *G. buergeri* was determined by PCR-based sequencing using fish versatile primers (Miya and Nishida 1999; Satoh et al. 2016). The mitogenome (DDBJ accession number: AP018347) was 16,529 bp in length with a more highly conserved structural organization (typical 37 genes and gene order) compared with that of other teleost fishes. The overall base composition of the L-strand was characterized by A (27.97%), C (29.39%), G (16.6%), and T (26.02%) and is similar to that observed in most other fishes (Satoh et al. 2016).

To accurately determine the phylogenetic position of glaucosomatids and intra-family relationships, a supermatrix analysis (de Queiroz and Gatesy 2007) was performed using 23 complete and 20 partial mitogenome sequences of percomorphs. The resultant supermatrix tree strongly supported the monophyly of Glaucosomatidae and revealed that the glaucosomatids were most closely related to Pempheridae (Figure 1). This close relationship observed in our study is supported by previous morphological analyses and nuclear gene studies (Tominaga 1986; Johnson 1993; Betancur et al. 2013; Thacker et al. 2015). Phylogenetic relationships within Glaucosomatidae were congruent with a previously published molecular study (Liu et al. 2010); *G. hebraicum* was placed as sister to a clade comprising *G. scapulare* and *G. buergeri*, and *G. magnificum* was placed at the most basal position within the glaucosomatids.

**Figure 1.**
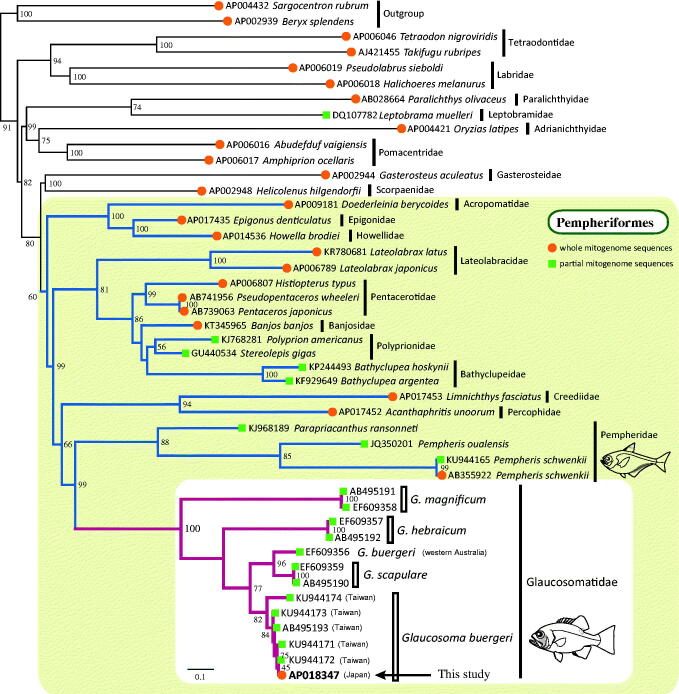
Supermatrix tree based on 23 complete and 20 partial mitogenome sequences of glaucosomatids and related species. Accession numbers are indicated before the species names. In the case of *G. buergeri* accessions, the location of collection is also indicated. Bootstrap values are indicated at each node. A backbone constraint tree was generated using complete mitogenome sequences (closed circles) by partitioned maximum-likelihood (ML) analysis. The analysis was conducted on a data matrix (13,439 positions) including all concatenated nucleotide sequences of the mitogenomes, except the ND6 gene. Gene sequences were aligned individually using the online version of MAFFT (http://mafft.cbrc.jp/alignment/server/; Katoh and Standley 2013), and ambiguous regions were trimmed using the online version of GBlocks with the least stringent settings (http://molevol.cmima.csic.es/castresana/Gblocks_server.html; Castresana 2000). Optimal partition model was determined using PartitionFinder ver 2 (Lanfear et al. 2017). Rapid bootstrap analyses were conducted with 1000 replications. Partitioned ML analyses were performed with RAxML-GUI ver. 1-5b1 (Silvestro and Michalak 2012) using the GTRGAMMAI nucleotide substitution model. The supermatrix tree was constructed based on 20 DNA barcode sequences (closed squares) that were prepared using the same methods as those used for the backbone tree with the GTRCAT model.

Results of phylogenetic analysis strongly supported the monophyly of each *Glaucosoma* species, with the exception of *G. buergeri* (EF609356) (Figure 1). *G. buergeri* (EF609356) from Western Australia clustered with *G. scapulare*, a species that is mainly distributed in Eastern Australia and has been rarely collected from Western Australia, according to the Atlas of Living Australia (https://www.ala.org.au/), an international biodiversity database. This inconsistency in data could have resulted from an introgression between the two species or, although unlikely, from mislabeling of samples. It is also possible that *G. buergeri* has intraspecies variation, wherein *G. buergeri* population in the northern hemisphere is genetically diverse from that in the southern hemisphere. To test these hypotheses, further investigations are required using a larger sample size collected from widely distributed geographical locations.
